# Nanotechnology in dermatologic therapy: clinical advances and emerging translational perspectives over the past decade^؆^

**DOI:** 10.1016/j.abd.2026.501427

**Published:** 2026-08-06

**Authors:** Clarissa Prati Bernardi Cogo, Mayra Ianhez, Renata Bertino, Anderson Alves Costa, Adilson Costa, Hélio Amante Miot

**Affiliations:** a Hospital São Lucas, Pontifícia Universidade Católica do Rio Grande do Sul, Porto Alegre, RS, Brazil; b Department of Dermatology, Hospital de Doenças Tropicais de Goiás, Goiânia, GO, Brazil; c Instituto de Educação Médica (Città), Universidade Estácio de Sá, Rio de Janeiro, RJ, Brazil; d Department of Dermatology, Hospital Ipiranga, São Paulo, SP, Brazil; e Instituto de Assistência Medica ao Servidor Público Estadual, São Paulo, SP, Brazil; f Department of Laboratory Medicine and Pathology, Mayo Clinic, Rochester, MN, United States of America; g Department of Infectology, Dermatology, Imaging Diagnosis and Radiotherapy, Faculdade de Medicina, Universidade Estadual Paulista, Botucatu, SP, Brazil

**Keywords:** Nanoparticles, Nanotechnology, Drug delivery systems, Topical drug administration

## Abstract

Nanotechnology has been revolutionizing science for at least five decades, with continuous expansion into various medical fields and the introduction of innovative strategies for the management of cutaneous conditions, particularly over the past decade. This article reviews the primary scientific evidence and recent technological advances regarding the application of nanoparticles in the management of skin diseases, highlighting their translational and regulatory relevance. The incorporation of nanocarriers has enabled the optimization and control of skin penetration, bioavailability, and drug and cosmetic active release, with improved safety and tolerability. These platforms have broadened therapeutic options for dermatoses and enhanced cosmetic outcomes in rejuvenation, hydration, and photoprotection. Nanoparticulate actives have demonstrated potential for improved efficacy and tolerability in selected clinical contexts, with greater patient adherence. In parallel, the use of nanotechnology in dermocosmetics and daily-use products has been growing exponentially, demanding greater regulatory standardization and strengthened oversight of manufacturing quality processes. Despite these advances, challenges remain regarding methodological reproducibility, physicochemical characterization, and traceability of nanomaterials. Furthermore, this critical narrative review acknowledges limitations, including heterogeneity in study designs and a lack of long-term data, which are necessary for a comprehensive understanding of the full impact of nanotechnology in dermatology. The integration of scientific innovation, regulation, and clinical practice is essential to consolidate the safe and effective use of nanoparticles in modern dermatology. Thus, nanotechnology represents a transition toward more precise, multifunctional, and personalized cutaneous therapies, with the potential to impact dermatologic public health and overall well-being.

## Fundamentals and historical context

Since Richard Feynman's theories on molecular manipulation in the 1950s, multiple lines of research have emerged to apply nanometric concepts.^1^ In medicine, radiology, oncology, and dermatology stand out as specialties that have significantly benefited from advances in nanometric materials for diagnostic and therapeutic applications of precision.^2^ Dermatology is a specialty particularly well-suited to the application of nanotechnology. Over the past decade, several platforms have already been employed to overcome the limitations of traditional topical formulations to treat diseases and cosmetic applications.^3^, ^4^

**Fig. 1:**
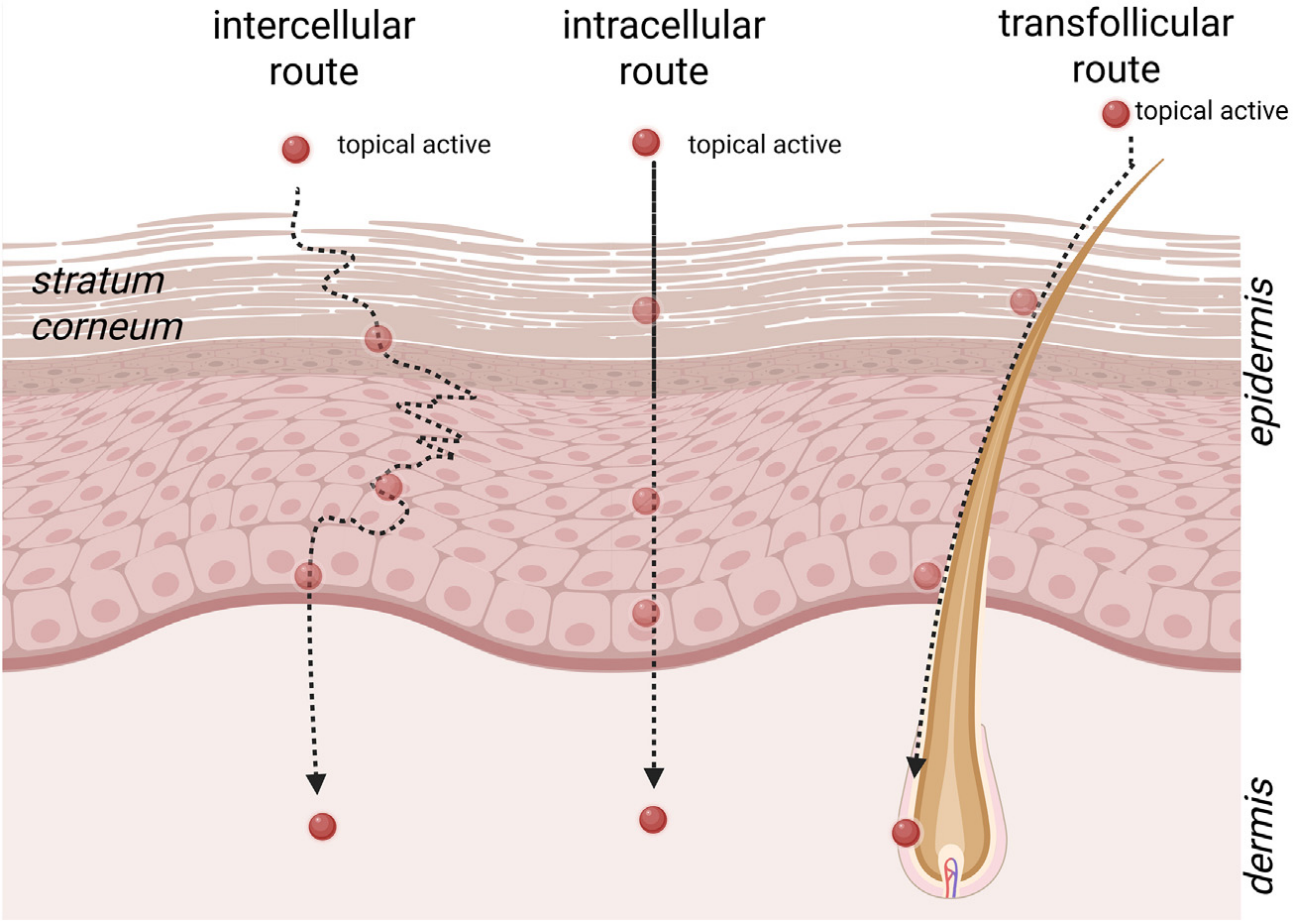
Schematic representation of the topical delivery pathways across human skin: the *intercellular* route (through the lipid matrix), the *transcellular* route (or intracellular ‒ across corneocytes), and the *transappendageal* route (follicular and eccrine sweat glands). Hair follicles act both as preferential entry points and as reservoirs that facilitate early penetration. Key determinants of permeation include molecular size, polarity, and the contribution of appendageal shunts.

The skin's stratum corneum acts as a selective barrier regulating exogenous substance flux or permeation by passive diffusion through three pathways: (through the cells), intercellular (between the cells), and transappendageal (through hair follicles and sweat glands), represented in Fig. 1. Penetration refers to the entry of the active ingredient into the superficial layers of the skin, whereas absorption implies its passage into the systemic circulation. Permeation efficiency depends on the active compound's physicochemical properties (molecular weight or size, lipophilicity, polarity), vehicle characteristics, skin condition, and application method.

While conventional topical formulations struggle to reach deeper skin layers, nanocarriers enhance permeation and local concentration of actives, reducing systemic absorption. By using nanocarriers to modify the physicochemical properties of active substances, it is possible to increase their affinity for the skin and promote controlled and targeted release. Thus, nanotechnology, combined with formulation engineering, holds the promise of greater therapeutic efficacy, reducing adverse effects, and improving product stability, potentially marking a milestone in developing safe and personalized cutaneous therapies.^5^

Formally, nanotechnology in topical products uses nanoscale structures (1‒1000 nm) to optimize the delivery, penetration, and controlled release of active ingredients.^6^ These nanocarriers, such as liposomes, solid lipid nanoparticles (SLNs), nanostructured lipid carriers (NLCs), dendrimers, and nanogels, encapsulate the active molecules, isolated or in associations. The choice of a specific nanocarrier is guided by design criteria such as particle size, surface charge, and surface ligands, which dictate their interaction with the skin.

Optimizing active ingredients in combined, multifunctional formulations can enhance patient adherence and lead to faster, more durable outcomes, making it a broad field for nanotechnology.^7^ This trend aligns with the growing knowledge of dermatological diseases, which is shifting disease management toward targeted therapies similar to those in clinical oncology.^8^ This includes developing new diagnostic and therapeutic solutions for topical and systemic use.^9^

Due to their chemical heterogeneity and wide application, nanoparticles have prompted international standardization efforts to ensure their safety, with guidance from agencies like the Food and Drug Administration (FDA) and the Brazilian Health Regulatory Agency (ANVISA).^10^, ^11^ However, the term “nano’ is often used inconsistently in topical products. It is crucial to understand that the functional concept of nanotechnology extends beyond mere particle size to include performance metrics, in essence, the bioavailability.^12^ Moreover, encapsulating active agents in nanomaterials can also make the nanocarrier itself a part of the therapeutic process.^13^

**Fig. 2:**
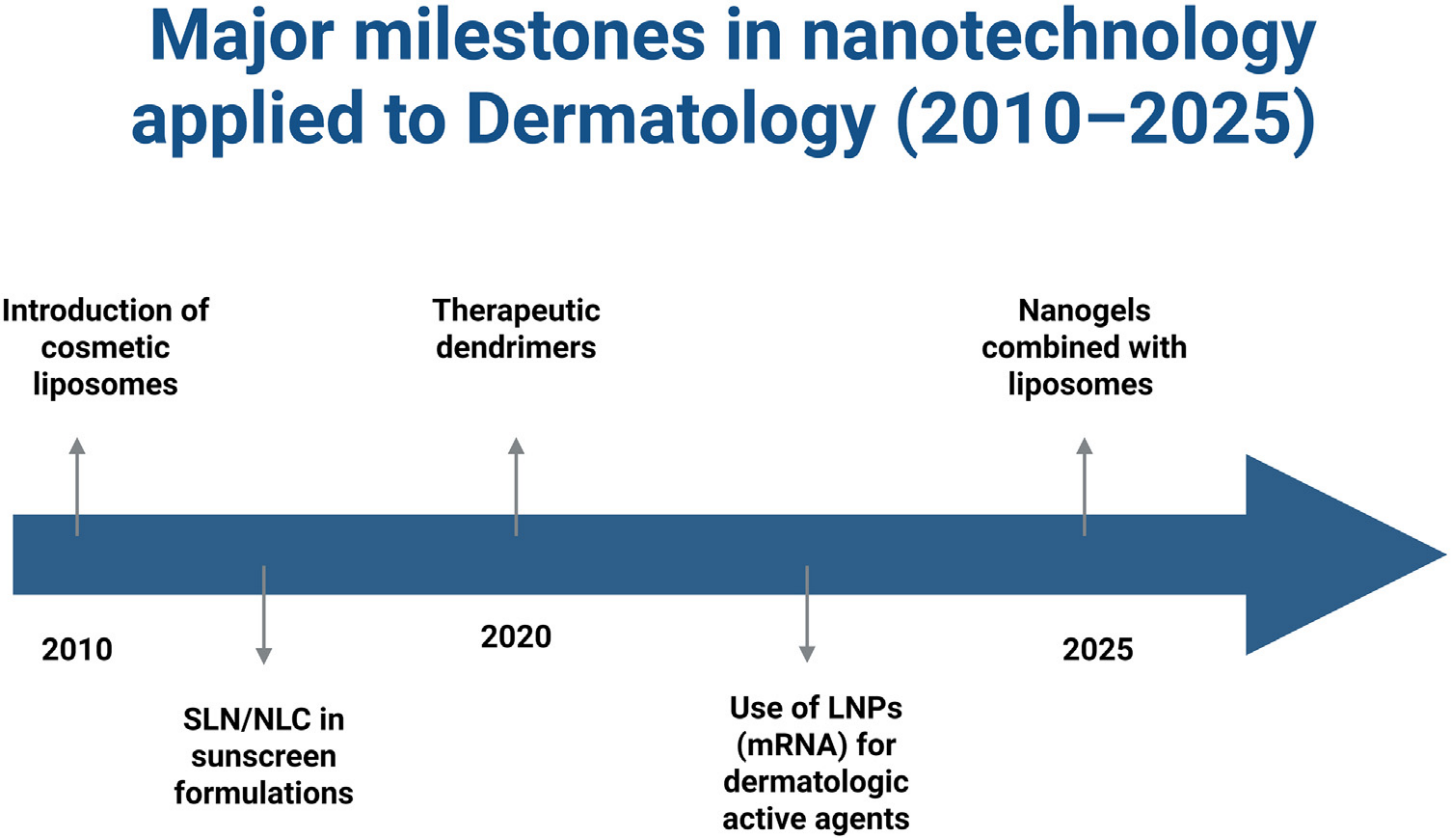
Timeline summarizing major milestones in the application of nanotechnology to dermatology from 2010 to 2025. Solid lipid nanoparticles and nanostructured lipid carriers (SLN/NLC), lipid nanoparticles (LNPs).

The ability of nanocarriers to precisely penetrate the skin and control the release of active ingredients has opened new therapeutic possibilities in both dermocosmetics and medicine.^14^ As illustrated in Fig. 2, this field has evolved significantly. The progression began with cosmetic liposomes for enhanced delivery, followed by solid lipid nanoparticles/nanostructured lipid carriers (SLNs/NLCs) around 2010, which improved SPF and user experience in sunscreens. By 2020, therapeutic dendrimers and mRNA-carrying lipid nanoparticles (LNPs) emerged for more precise treatments. The projected development of nanogel-liposome systems by 2025 exemplifies the ongoing trend toward more sophisticated, multifunctional carriers with enhanced stability and targeted release.

Up-to-date knowledge of nanotechnology is essential for dermatologists, as clinical practice demands critical appraisal of evidence, toxicity monitoring, and regulatory compliance. This critical narrative review synthesizes advancements in skin-applied nanotechnology over the past decade, discusses emerging trends, and contextualizes findings within the broader literature. The following sections bridge these lab discoveries with emerging clinical applications, acknowledging current evidence limitations.

This narrative review was based on a structured search of PubMed, Scopus, and Web of Science databases between 2015 and 2025 using the terms ‘nanoparticles’, ‘dermatology’, ‘topical delivery’, and ‘nanocarriers’. Priority was given to human clinical studies and translational research, while relevant preclinical studies were included when clinically informative.

## Nanoplatforms for dermatology

The skin functions as a dynamic biological barrier, posing challenges for the delivery of specific therapeutic agents due to factors such as stability, polarity, solubility, or molecular weight. Nanotechnology offers solutions to overcome these permeation barriers, increase the bioavailability of lipophilic drugs, reduce irritation, and enable localized release.^15^

Products composed of nanoparticles with heterogeneous size distributions can result in unpredictable behavior: larger particles remain on the surface, medium-sized particles penetrate the epidermis, and smaller particles reach the superficial dermis, where systemic absorption occurs.

**Fig. 3:**
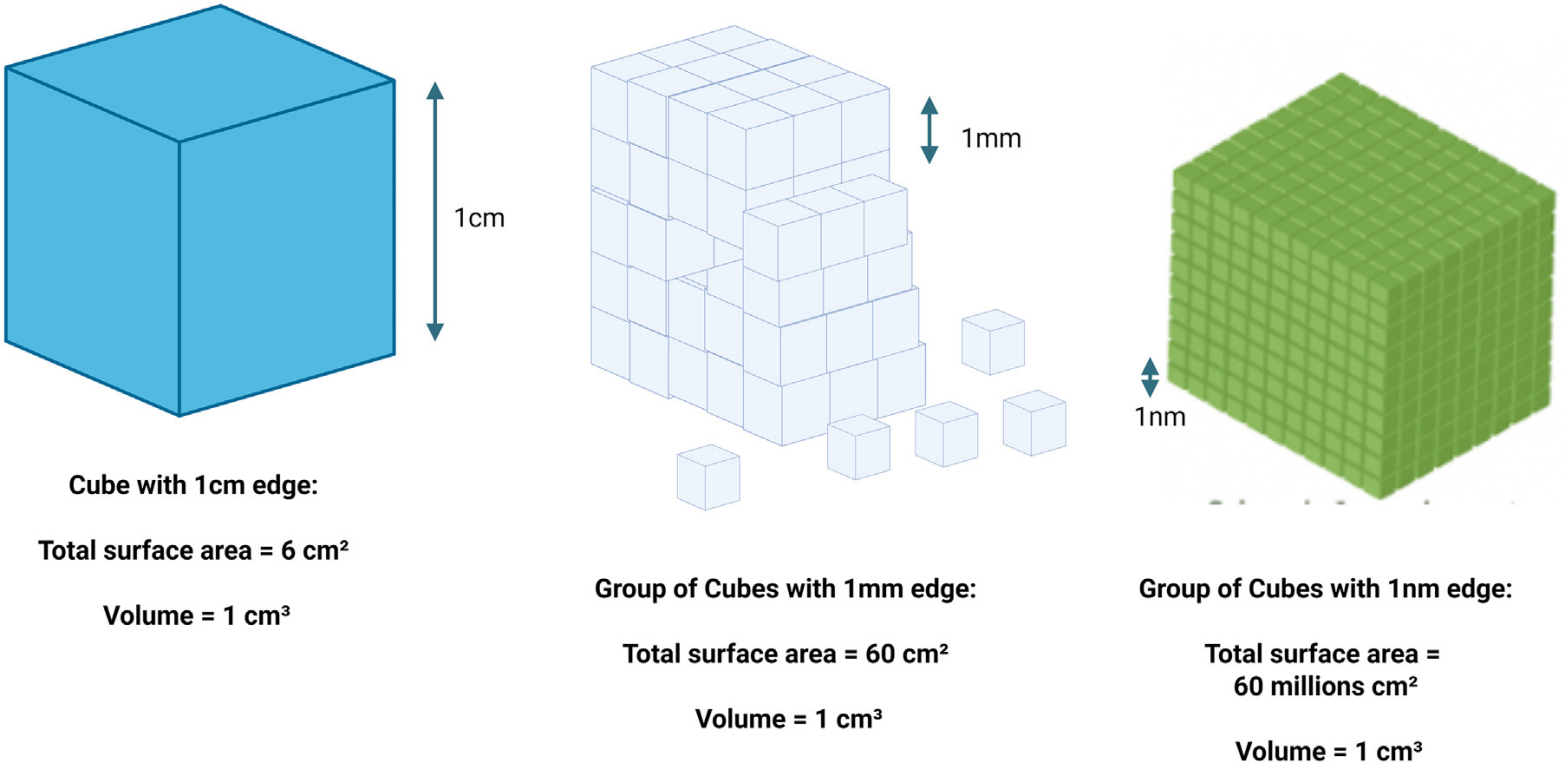
Schematic representation of the magnitude of the total surface area when the same material volume (1 cm^3^) is subdivided into cubes at the nanoscale.

**Fig. 4:**
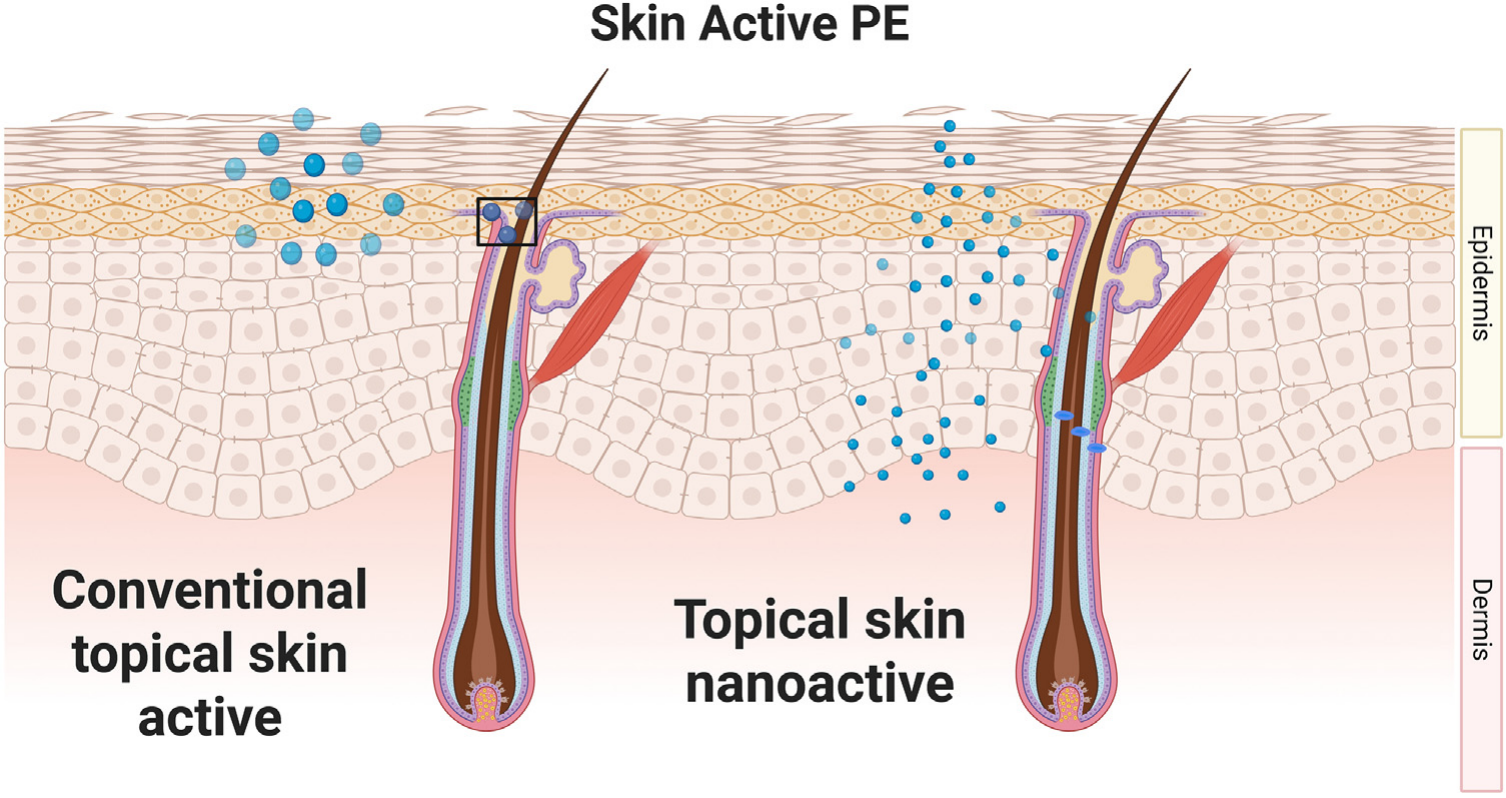
Illustration comparing the area of coverage, precision, and uniformity of distribution of a conventional topical active versus a nano-enabled active. The nanoformulation achieves improved permeation across the stratum corneum and enhanced appendageal delivery, particularly through hair follicles and the pilosebaceous unit, resulting in greater early deposition and more homogeneous distribution within the skin.

**Fig. 5:**
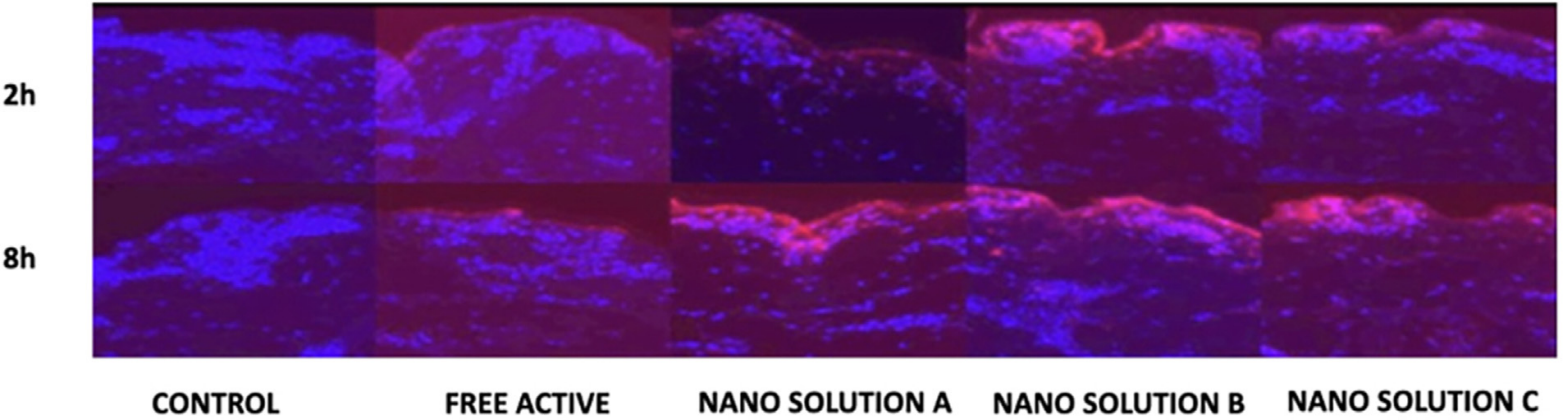
Skin permeation of nanoencapsulated solutions of retinyl palmitate, ascorbic acid, and bakuchiol (NS01–NS03) versus non-encapsulated actives in a Franz diffusion cell (confocal microscopy at 2 h and 8 h). Red indicates active ingredients. Unpublished data on file; study performed by Bettech, Rio Grande do Sul, Brazil. The Franz diffusion data shown are derived from previously published experimental studies and are presented for illustrative purposes.

Beyond particle size, the high surface-area-to-volume ratio of nanocarriers enables more homogeneous tissue interaction. Fig. 3, Fig. 4 illustrate how subdividing a given volume while maintaining proportions increases surface area and precision of active ingredient incorporation during skin permeation. Fig. 5 demonstrates this principle through a Franz diffusion cell experiment using confocal fluorescence microscopy. At 2- and 8 -hs, vehicle control and non-encapsulated active showed minimal fluorescence, while three Nanosolutions (NS01–NS03), retinyl palmitate in polymeric, ascorbic acid in liposomal, and bakuchiol in hexosomal nanocarriers, produced markedly higher and deeper epidermal signals. At eight hours, NS01, NS02, and NS03 achieved approximately 2-fold, 2.4-fold, and 2-fold higher permeation than free actives, respectively, with the liposomal system (NS02) showing the most pronounced enhancement in cutaneous penetration and retention. (Unpublished data on file; study performed by Bettech, Rio Grande do Sul, Brazil).

Nanoparticles must be deliberately designed considering physicochemical and biological parameters that determine performance and safety, including biopersistence, biocompatibility, and controlled degradation, which vary by material and intended use.^16^, ^17^ The diversity of formats: lipid-based, polymeric, metallic, or hybrid, broadens their applications. However, no single nanoparticle type is universally recognized as most effective or safest for all dermatologic indications due to complex nanomaterial-cutaneous barrier interactions.^18^ Critical thresholds serve as practical formulation targets: ζ potential of ±30 mV for colloidal stability and polydispersity index (PDI) < 0.2 for particle size uniformity.^18^

Effective nanoparticle development requires optimization of structural properties: size, morphology, surface charge, and chemical composition, aligned with intended use. In dermocosmetics, this precision engineering creates multifunctional systems that serve as both carriers and nanoactive components, enhancing active ingredient efficacy, prolonging release, improving stability, and exerting direct effects on hydration, epidermal barrier restoration, and cutaneous modulation.^18^ Table 1 summarizes cutaneous layers, their physiological barriers, and implications for rational nanomaterial design in topical and transdermal delivery. Selecting nanocarriers with specific structural characteristics enables targeted outcomes, such as combining unsaturated lipids to improve hydrophobic vitamin delivery.

**Table 1:** Overview of skin layers, key physiological barriers, and implications for nanomaterial design in cutaneous delivery.

Skin layer/structure	Key structural and physiological features	Dominant barrier characteristics	Implications for nanomaterial design
Stratum corneum	Cornified envelope of dead keratinocytes embedded in a highly ordered lipid matrix; low water content; acidic pH	Principal barrier to diffusion; very low permeability, especially for hydrophilic and high-molecular-weight compounds	Favors small (< 100–200 nm), often lipid-based carriers; design of occlusive or film-forming systems, penetration enhancers, and deformable vesicles to transiently modulate barrier resistance while avoiding disruption of integrity
Viable epidermis (excluding stratum corneum)	Living keratinocytes, melanocytes, Langerhans cells; active metabolic and immunologic environment	Selective permeability, active metabolism, and immune surveillance; risk of irritation or sensitization	Requires controlled permeation and local retention; design of nanocarriers with tuned release profiles and surface properties to minimize immunogenicity while enabling targeting of keratinocytes and melanocytes
Dermis	Fibroblasts, collagen and elastin network, vasculature, lymphatics, nerves, adnexal structures	Dense extracellular matrix and vascularization; potential for systemic distribution once barrier is crossed	Supports design of nanocarriers for localized delivery to fibroblasts and vasculature (e.g., anti-inflammatory, pro-regenerative agents) or, when desired, for transdermal/systemic delivery using microneedles or other minimally invasive technologies
Skin appendages (hair follicles, sebaceous and sweat glands)	Discrete invaginations crossing the stratum corneum; reservoirs of sebum and microbiota	Provide preferential shunt pathways and long-term depots for topically applied particles	Enable follicularly targeted nanocarriers (e.g., 50–300 nm), optimized for accumulation in pilosebaceous units in acne, alopecia, and reservoir-based delivery systems
Hypodermis (subcutaneous tissue)	Adipose tissue with connective septa, vasculature and nerves; mechanical cushioning	Not a primary barrier to diffusion; acts mainly as a depot for injected substances	Relevant mainly for injectable or scaffold-based nanoplatforms; design of long-acting depots and tissue-regenerative systems rather than classic topical formulations

Note: This table summarizes the structural and functional characteristics of each skin layer relevant to nanomaterial design. The implications listed represent current best practices in formulation design and are subject to ongoing optimization as new evidence emerges.

**Fig. 6:**
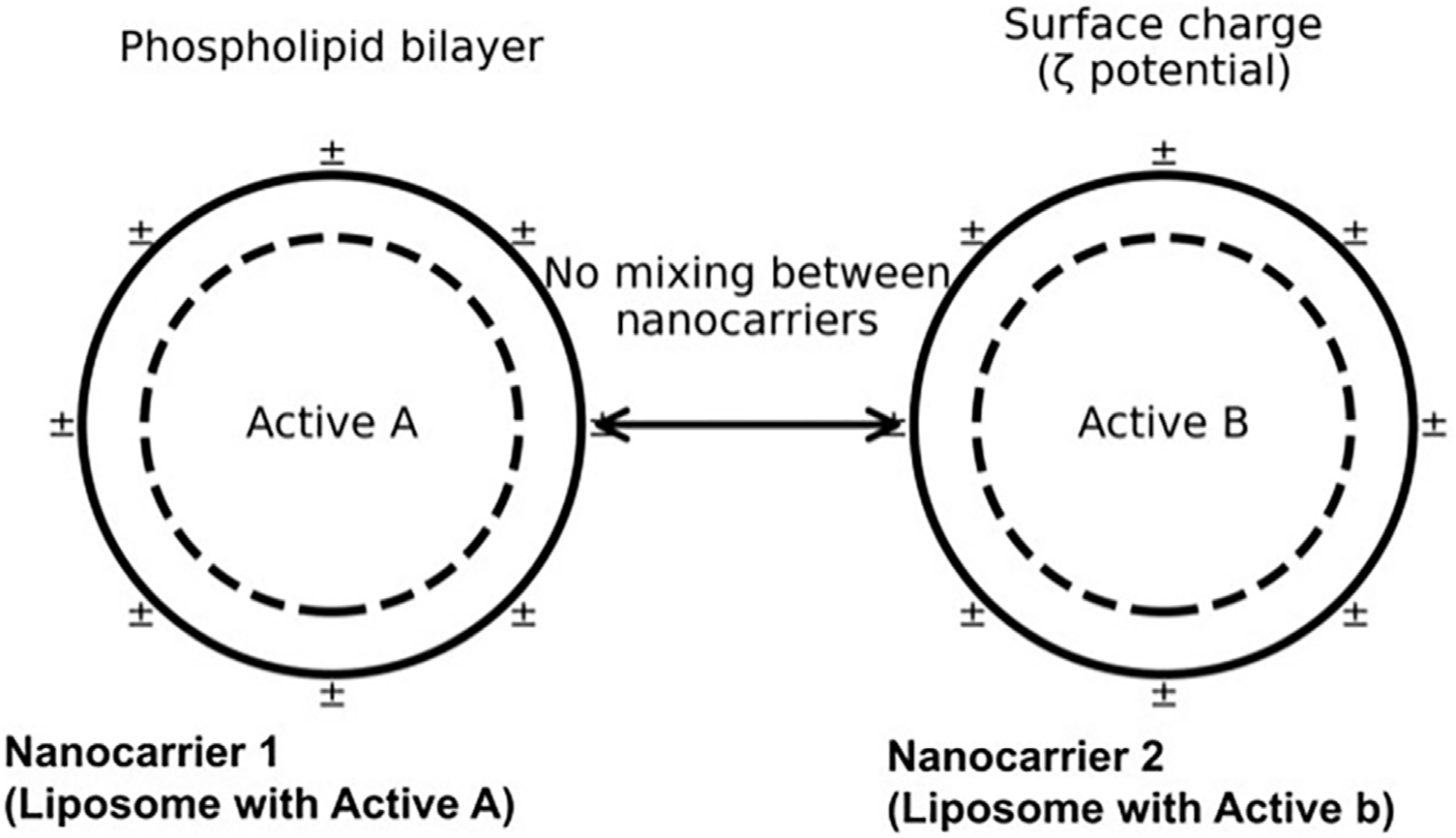
Schematic representation of liposomal nanocarriers enabling multi-active encapsulation in a single topical formulation.

Nanotechnology-based products are generally more effective than equivalent active mixtures because physical separation of actives minimizes chemical interactions, increases capacity for multiple sensitive ingredients, and improves stability and performance,^19^, ^20^, ^21^ as illustrated in Fig. 6, where each nanocarrier (phospholipid bilayer with aqueous core and ζ potential) carries a distinct active.

**Fig. 7:**
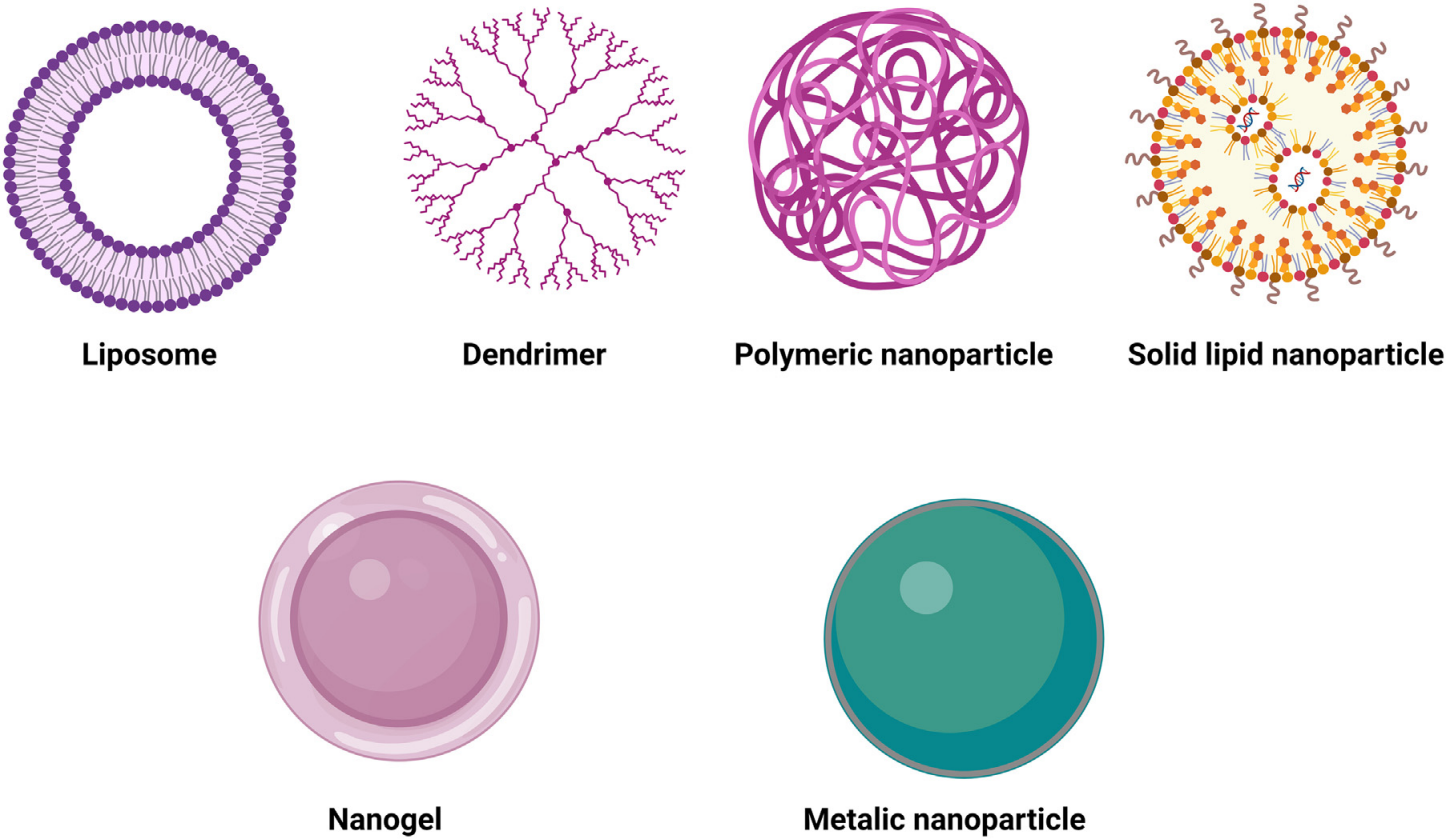
Architectural illustration of the main nanocarriers used in dermatology.

Dermatologic nanocarriers include traditional liposomes, solid lipid nanoparticles, nanoemulsions (50–200 nm),^14^, ^22^ as well as polymeric nanoparticles, dendrimers, nanogels, gold and silica nanoparticles, and nanostructured hydrogels. Table 2 summarizes their main characteristics and dermatologic applications,^23^, ^24^, ^25^, ^26^, ^27^, ^28^, ^29^, ^30^, ^31^, ^32^ while Fig. 7 depicts their architectural representations. Table 3 presents representative human clinical studies evaluating topical nano-enabled formulations with explicitly described nanocarriers and clearly defined dermatologic indications from the past 10-years, identified through a targeted narrative search of the biomedical literature.

**Table 2:** Description of some of the nanoplatforms for topical dermatologic treatment.

Nanoparticle	Key characteristics	Topical use
Liposomes^23^, ^24^	Phospholipid vesicles (bilayer) that encapsulate hydrophilic drugs (in the aqueous core) and lipophilic drugs (in the membrane)	Vitamin C, vitamin E, hyaluronic acid, coenzyme Q10, corticosteroids; actives used in anti-aging, skin-lightening, and anti-inflammatory formulations
Niosomes^25^	Vesicles composed of non-ionic (non-phospholipid) surfactants	Retinol, antioxidants, minoxidil in hair formulations, and anti-aging actives
Polymeric nanoparticles^26^	Biodegradable polymer matrix/core-shell systems (PLA/PLGA) or cationic polymers (chitosan)	Vitamin C, UV filters, antibiotics, antimicrobial peptides; used to increase stability and skin penetration
Metal nanoparticles^27^	Inorganic core (Au, Ag, ZnO, TiO_2_)	ZnO and TiO_2_ in sunscreens; Ag in antimicrobial creams; Au in dermatologic phototherapy
Dendrimers^28^	Highly branched macromolecules with multiple terminal groups	Carriers for antimicrobial peptides, antioxidants, and other bioactive molecules in topical formulations
Solid lipid nanoparticles (SLN) and Nanostructured Lipid Carriers (NLC)^29^	Solid lipid core (SLN) vs. combined solid + liquid lipid core (NLC)	Retinol, coenzyme Q10, UV filters, antifungals, skin-lightening agents; used to enhance cutaneous penetration
Protein nanocapsules^30^, ^31^	Core/complex in which proteins (e.g., albumin) form the matrix/carrier	Experimental formulations containing antioxidants and plant extracts for skin applications
Lipid nanoparticles for RNA (LNPs)^32^	Mixture of ionizable and helper lipids that encapsulates mRNA/siRNA	Formulations under development for antioxidant actives and anti-aging peptides

PLA, Polylactic Acid; PLGA, Poly (Lactic-co-Glycolic) Acid; Au, Gold; Ag, Silver; ZnO, Zinc Oxide; TiO_2_, Titanium Dioxide.

**Table 3:** Examples of nanotechnology-based topical systems in dermatology with human clinical studies over the past 10-years.

Nanocarrier	Active Ingredient	Indication	Study Design
Tea tree oil nanoemulsion (TTO)^36^	Adapalene 0.1%	Acne vulgaris	Triple-blind, randomized clinical trial (100 patients), 12-weeks
Nanoliposomes (gel)^37^	Amphotericin B 0.4%	Cutaneous leishmaniasis	Open-label pilot clinical study
BF-200 ALA nanoemulsion (Ameluz®)^38^	5-Aminolevulinic Acid (ALA) 7.8%	Superficial basal cell carcinoma (and extensive data in actinic keratosis)	Phase III randomized non-inferiority clinical trial
Oleosome-based nanoformulation of methylene blue (MBSD)^39^	Methylene blue + salicylic acid (photodynamic therapy)	Truncal acne	Open-label clinical study (24 patients), 5 weekly sessions
Niosomes (topical solution)^40^	Clindamycin phosphate 1%	Acne vulgaris	Randomized, double-blind clinical trial (100 patients)
Nanostructured lipid carriers (NLC-TRE) in gel/cream^41^	Tretinoin 0.05%	Mild-to-moderate acne vulgaris	Double-blind, split-face randomized clinical study comparing NLC-tretinoin with conventional 0.05% tretinoin cream
Tretinoin nanogel (oil-in-water nanoemulsion gel)^42^	Tretinoin 0.025%	Facial acne vulgaris	Randomized, active-controlled, multicentre, phase IV clinical trial comparing 0.025% tretinoin nanogel with conventional 0.025% tretinoin gel

Liposomes are spherical vesicles composed of phospholipid bilayers and an aqueous core, structurally similar to cell membranes,^33^ with variable sizes and morphologies (unilamellar, multilamellar, or multivesicular vesicles). Owing to this conformation, they can carry both hydrophilic and lipophilic actives individually or in combination, protecting sensitive substances, reducing incompatibilities between co-formulated actives, and enhancing penetration and stability.^34^ This is achieved because liposomal formulations concentrate within the stratum corneum and release actives in a controlled manner, thereby reducing cumulative drug concentration and adverse effects while maintaining therapeutic efficacy.^35^, ^36^, ^37^, ^38^, ^39^, ^40^, ^41^, ^42^

The total loading capacity of a liposome is variable and influenced by its characteristics, such as vesicle size and the presence of unilamellar or multilamellar phospholipid bilayers.^43^, ^44^ Therefore, the final concentration of actives in each nanocapsule depends not only on the amount of lipids and internal volume, but also on the encapsulation technology, which tends to be proprietary to each developer. This technology is crucial for ensuring stable carrier permeation and the bioavailability of actives, thereby creating truly multifunctional nanoplatforms.

Recent nanotechnology manipulations have involved combining carriers, taking advantage of specific characteristics that complement one another within a single release system. This approach enhances cutaneous permeation, as different types of nanoparticles act synergistically to modulate the stratum corneum barrier. Hybrid systems offer multifunctionality, allowing a single formulation to exert multiple actions such as protection, stabilization of actives, and multilevel controlled release. These advances expand the therapeutic and cosmetic potential of nanocarriers, making them promising platforms in aesthetic dermatology and the treatment of cutaneous and systemic diseases.^21^

Liposomes are being optimized as new combinations of actives and individualized treatment needs emerge. A new generation of deformable nanovesicles, transfersomes, has acquired structural adaptability by incorporating edge activators, such as sodium deoxycholate, into the phospholipid bilayer.^45^, ^46^ These carriers have reached a new level of topical delivery performance through their high flexibility, which allows them to deform and permeate the stratum corneum into the viable epidermis. This enhances outcomes such as those observed with growth factors for wound healing in animal models, where results were 60% superior to the usual active.^22^ Moreover, the association of gel technologies with liposomes has gained prominence in optimizing dermatologic treatments, as in the case of nano- and microgels.^47^, ^48^, ^49^

## Nanotechnology in cosmetics

Brazil is the fourth-largest cosmetics market in the world, with an estimated value of US$22.9 billion in 2022.^50^ Currently, demand for cosmetics with improved clinical and sensorial performance is directly related to innovation and research applied to formulations, which increasingly focus on enhancing the stability and efficacy of active ingredients.^7^ Consumers are increasingly concerned with appearance and well-being, with growing demand for products focused on personalization and skin health, reflecting a market increase of approximately 6% in 2021.^50^

Dermocosmetics represent an important frontier of skin-applied nanotechnology, combining cosmetic benefits with therapeutic effects. In nanostructured dermocosmetic formulations, cosmetic actives (antioxidants, UV filters, moisturizers, vitamins, vitamin A metabolites) are encapsulated in liposomes, SLN, and NLC to enhance stability, ensure uniform application, enable controlled release, improve penetration of lipophilic compounds, and reduce local irritation, which is particularly relevant in products intended for sensitive skin, skin prone to dyschromias (such as darker phototypes), elderly individuals, or patients with chronic inflammatory conditions. Clinical evidence underscores the need to balance cosmetic improvement with long-term safety and ensure regulatory oversight and clear communication with patients about expectations and limitations.^9^, ^51^, ^52^

Nanotechnology significantly enhances skin cleansing outcomes. Effective skin cleansing is essential for maintaining cutaneous homeostasis, as the stratum corneum lipid barrier accumulates sebum, sweat, pollutants, and microbes that can trigger inflammation. Traditional surfactant-based cleansers often compromise barrier integrity, leading to dryness, irritation, and microbiome imbalance. Nanotechnology-enabled cleansing systems, including oil-in-water nanoemulsions, functionalized silica nanoparticles, polymeric micelles, and layered double hydroxides, leverage high surface-area-to-volume ratios to selectively adsorb lipophilic debris and pollutants while minimizing barrier disruption. Stimuli-responsive nanomaterials that respond to environmental cues, such as pH or oxidative stress, can provide on-demand release of active ingredients and gentle exfoliation, offering a sophisticated approach to cleansing sensitive or disease-prone skin.^51^, ^52^

Optimal skin hydration is essential for barrier preservation in healthy skin and symptom control in chronic situations such as atopic dermatitis, psoriasis, and xerosis in the elderly. Nanostructured moisturizers, including nanoemulsions, liposomes, and lipid-based carriers loaded with humectants (e.g., glycerol, hyaluronic acid), emollients, and barrier-repair lipids, enhance water retention and improve stratum corneum plasticity. These systems exhibit improved penetration into upper epidermal layers and prolonged residence time, resulting in greater patient adherence, tolerance, and more durable clinical benefits.^51^, ^52^

Topical nanocosmetic formulations offer advantages such as improved skin hydration, enhanced UV protection, occlusive effects, and greater product stability. Lipid-based nanocarriers, including SLNs and NLCs, form occlusive films that reduce transepidermal water loss and increase hydration while improving active ingredient dispersion and stability.^53^ Nanoemulsions used in moisturizing formulations significantly enhance skin hydration through occlusive properties and optimized droplet-size distributions.^54^, ^55^ In photoprotection, inorganic nanoparticle-based filters such as nano-sized titanium dioxide and zinc oxide provide broad-spectrum, photostable UV protection with improved cosmetic acceptability compared with larger particles.^56^, ^57^, ^58^ Nanocarriers confer increased physical and chemical stability to labile cosmetic actives, enhanced skin penetration, and sustained release, translating into superior product performance.^58^ However, nanoformulation in dermocosmetics raises essential questions regarding manufacturing quality, labelling, and consumer transparency. Regulatory approaches differ between countries, involving criteria related to safety, stability, and compatibility with active ingredients. Supplementary Table 1 presents examples of dermocosmetic products with declared nanotechnology use over the past five years.

## Nanotechnology-enabled anti-aging, photoaging, and management of specific conditions

Skin aging results from intrinsic changes and UV-induced photoaging, leading to oxidative stress, collagen degradation, DNA damage, chronic inflammation, and pigmentary alterations. Nanotechnology-enabled anti-aging formulations provide a platform to counteract these pathways by enhancing targeted delivery of retinoids, peptides, antioxidants, and growth factors to specific skin layers, while improving stability and enabling controlled release. By co-encapsulating brightening agents, barrier-repair lipids, and hydrating components, multifunctional nanosystems can simultaneously modulate oxidative stress, support extracellular matrix remodeling, and improve skin tone and texture, offering a holistic approach to facial rejuvenation.^9^, ^51^, ^59^

For melasma, solid lipid nanoparticles and nanostructured lipid carriers enhance cutaneous penetration of depigmenting agents (azelaic acid and hydroquinone) while providing sustained release and reducing adverse effects.^60^ Antioxidants encapsulated in nanostructured systems modulate melanogenesis and protect against UV-induced damage, contributing to hyperpigmentation control.^61^

Tranexamic acid is a promising depigmenting agent for melasma and post-inflammatory hyperpigmentation, but low cutaneous permeation limits conventional formulation efficacy. Lipid vesicular systems (liposomes and ethosomes) enable Tranexamic Acid (TXA) encapsulation, promoting controlled release, stability, and prolonged skin penetration. Lipid vesicles significantly increase drug deposition in the epidermis and improve clinical response compared with traditional formulations.^62^ Guo et al. (2023) reported marked melasma improvement in phototypes III–IV with 0.5% TXA ethosomes, without adverse events.^63^ The combination of tyrosinase inhibition, TXA's antiangiogenic effect, and nanocarrier performance represents a significant advance in hyperpigmentation treatment safety and efficacy, especially in pigmented skin types.^64^

Lipid nanoparticles, polymeric carriers, and liposomes are widely used to encapsulate actives such as vitamin C, retinol, and hyaluronic acid, protecting them from oxidation and degradation while enhancing cutaneous penetration and bioavailability. Retinol-loaded lipid nanocarriers and nanostructured lipid carriers show markedly improved chemical stability and approximately two-fold higher skin deposition compared with non-encapsulated retinol.^65^, ^66^ Elastic and cationic liposomal systems increase ascorbic acid penetration through the stratum corneum into deeper epidermal layers, maintaining antioxidant activity.^67^, ^68^ Hyaluronic-acid-based nanoparticles and nanogels support sustained water binding and improved barrier function.^69^ Clinical and translational studies show enhanced skin hydration, homogeneous pigmentation, and reduced fine lines and wrinkles, particularly with nanoencapsulated vitamin C or retinoids combined with supportive moisturizing systems. Physical enhancement methods such as microneedling and iontophoresis combined with nanoparticle carriers optimize targeted delivery to specific cutaneous compartments, maximizing efficacy while limiting systemic exposure.^70^

It is important to note that a substantial proportion of topical products currently marketed as “exosome” formulations are, in practice, conventional liposomal systems containing plant-derived or synthetic actives rather than true biological exosomes.^71^ By definition, exosomes are nanoscale extracellular vesicles of endosomal origin that carry complex biological cargo, including proteins, lipids, messenger RNA, microRNA, and growth factors, released by living cells and capable of mediating intercellular communication.^72^, ^73^ In contrast, many so-called exosome creams and serums available in the cosmetic market are essentially phytocosmetic ingredients or peptides encapsulated in liposomes, without demonstrable extracellular vesicle structure or validated exosomal markers. This discrepancy between the biological concept of exosomes and the actual composition of most commercial “exosome” products underscores the need for precise terminology, transparent labelling, and critical appraisal of marketing claims when interpreting the purported regenerative or rejuvenating effects of these topical formulations.

## Nanotechnology in dermatologic diseases

In topical treatment, nanocarriers such as nanoemulsions, liposomes, polymeric nanoparticles, nanogels, and nanocrystals are used to overcome the epidermal barrier, thereby increasing the bioavailability of active agents in the deeper layers of the skin. This is particularly relevant in conditions such as atopic dermatitis, psoriasis, acne, vitiligo, alopecia, skin aging, and skin cancer, in which therapeutic efficacy depends on the precise and sustained delivery of agents.^13^ Examples of active ingredients formulated in nanoparticles for dermatologic diseases are presented in Table 4.

**Table 4:** Application of nanotechnology across dermatologic diseases.

Dermatologic disease	Type of nanotechnology	Active agent	Objective
Acne	Liposomes	Azelaic acid	Improved tolerability
Rosacea	Liposomes	Azelaic acid	Improved tolerability
Hyperpigmentation	‒	Benzoyl peroxide	Improved permeation
Vitiligo	Liposomes	‒	‒
Vitiligo	Polymeric nanoparticles	Tacrolimus	Reduce systemic exposure
Vitiligo	Polymeric nanoparticles	Tofacitinib	Reduce systemic exposure
Vitiligo	‒	*Polypodium leucotomos*	Reduce systemic exposure
Atopic dermatitis	Caprolactone nanoparticles	Hydrocortisone	Improved efficacy, permeation, and safety
Atopic dermatitis	Solid lipid nanoparticles	Betamethasone valerate	Improved efficacy, permeation, and safety
Atopic dermatitis	Nanoemulsion	Clobetasol propionate	Improved efficacy, permeation, and safety
Tuberous sclerosis (angiofibromas)	‒	Sirolimus	Mild adverse effects; no systemic activity
Dermatophytoses	Nanosponges	Ciclopirox olamine	Increased efficacy; greater sustained release
Psoriasis – plaque	Liposomes and polymeric nanoparticles	Calcipotriol	Penetration into thickened skin layers and sustained release
Psoriasis – plaque	Liposomes modified with DSPE-PEG	Calcipotriol	Improved penetration with small vesicles
Psoriasis – plaque	Cationic liposomes	Cyclosporine	Reduction of proinflammatory cytokines (TNF-α, IL-17, IL-22) and symptoms
Psoriasis – plaque	Liposomes	Glabridin; ibrutinib/curcumin	Reduced psoriasis severity and inflammatory cytokines
Psoriasis – guttate	Nanogels and dendrimers	‒	Sustained release and anti-inflammatory properties
Psoriasis – pustular	Solid lipid nanoparticles (SLN) and nanostructured lipid carriers (NLC)	Methotrexate; leflunomide	Amphiphilic behavior (hydro-/lipophilic) and moisturizing effect
Psoriasis – inverse	Nanoemulsions	‒	Favors penetration in skin folds

DSPE-PEG (1,2-Distearoyl-sn-glycero-3-Phosphoethanolamine-N-Polyethylene-glycol). NLC, Nanostructured Lipid Carriers; SLN, Solid Lipid Nanoparticles.

Inflammatory disorders such as acne and rosacea are prominent targets for nanotechnology-based topical management. Azelaic acid, for instance, has a long history of use and a broad spectrum of beneficial effects in these conditions, including antibacterial and anti-inflammatory actions, reduction of hyperkeratinization, and improvement of dyschromia, with a relatively gentle tolerability profile. More recently, a liposomal foam formulation of azelaic acid has shown favorable outcomes, with bioavailable distribution across all skin layers^35^ and clinically proven efficacy compared with placebo and benzoyl peroxide in the management of hyperpigmentation.

An ethosomal gel containing clindamycin and salicylic acid reduced acne lesions more rapidly than conventional formulations and was better tolerated by patients, likely because the system released the actives gradually within the pilosebaceous unit, thereby avoiding peak epidermal irritation.^74^ In addition, various nanosystems (liposomes, nanoemulsions) have already been tested to deliver benzoyl peroxide, adapalene, tazarotene, and other anti-acne agents, demonstrating less dryness and desquamation of the skin compared with the traditional formulations.^75^, ^76^

In the context of vitiligo, nanotechnology has been explored through liposomal and polymeric nanoparticle delivery systems, which have shown efficacy in promoting repigmentation and modulating the local immune response. Recently, an *in vitro* study demonstrated greater membrane retention of nanoencapsulated tacrolimus, potentially helping optimize the in vivo drug dose while minimizing systemic exposure.^77^ Similar strategies have been investigated for the cutaneous delivery of tofacitinib, a Janus kinase inhibitor with several systemic indications, whose hematologic and other adverse effects can limit its use in specific patient profiles.^78^, ^79^

Moreover, nanostructured systems containing antioxidants, such as *Polypodium leucotomos* extracts and other plant-derived compounds, have shown potential to protect melanocytes and promote repigmentation, acting as adjuvants to phototherapy.^80^ Nanoparticles with catalase-like activity (PAPLAL) have been used to combat oxidative stress implicated in melanocyte destruction. Preliminary human results indicated repigmentation in refractory cases.^81^ Another promising strategy is the use of ultradeformable nanovesicles loaded with psoralen and antioxidants (resveratrol) to optimize PUVA phototherapy: *in vitro* and animal studies have shown increased melanogenesis and enhanced antioxidant protection in the skin with these nanocarriers.^82^

For atopic dermatitis, in addition to the new systemic medications already approved and under development for moderate-to-severe disease, there remains a clear need for topical management of flare-ups and of milder cases. In this setting, the use of topical corticosteroids and immunomodulators remains an excellent option.^83^ Ointment formulations of hydrocortisone in caprolactone nanoparticles, betamethasone valerate homogenized in solid lipid nanoparticles, and clobetasol propionate in nanoemulsions have demonstrated comparable efficacy results and an acceptable adverse-effect profile when compared with conventional presentations.^84^, ^85^, ^86^ Furthermore, nanotechnology has already been adapted to tacrolimus ointment, providing improved permeation at lower doses and potentially optimizing treatment costs, whether through its combination with chitosan and nicotinamide^87^ or through the use of microemulsions,^88^ which achieved at least threefold greater *in vivo* penetration. In addition, polymeric nanoparticles associated with hyaluronic acid have also shown superior controlled release when compared with traditional formulations.

Although they are not considered first-line agents for topical management of eczema, antihistamines are also beginning to appear in nanocarrier-based presentations, such as levocetirizine in a chitosan-based gel, which has shown beneficial effects on erythema and pruritus.^89^ The development of topical nanoformulations of methotrexate, which exerts a potent inhibitory effect on proinflammatory cytokines, has likewise contributed to improved control of the immune response.^90^

Patients with rare and challenging diseases have also benefited from research and development of nanocarrier-based actives in independent centers. Spanish authors reported the results of a prospective observational study that included 11 patients with facial angiofibromas associated with tuberous sclerosis who were treated with 0.4% liposomal sirolimus for 24-weeks.^91^ These lesions are known to have a significant impact on self-esteem and are often managed in parallel with other disease-related complications. The novel formulation was well tolerated, with only mild adverse effects and no evidence of systemic absorption, and was associated with a reduction in lesion burden and high patient satisfaction.

The activity of traditional topical antifungals can be compromised by particle size and low solubility, characteristics that nanotechnology effectively addresses, as in the recently described incorporation of ciclopirox olamine into nanosponges. The authors demonstrated superior antifungal activity compared with the isolated drug and commercially available topical creams, as well as sustained *in vitro* release.^92^

A vast field of research on the use of nanocarriers involves the management of psoriasis. Nanoparticles represent promising strategies for treating this disease because they deliver drugs directly to lesions, thereby increasing efficacy and reducing adverse effects.^93^ Moreover, depending on the clinical subtype of psoriasis, certain technologies may be more appropriate than others, as can be seen in Table 4.

The use of liposomes in the topical treatment of psoriasis has also shown promise, improving formulation stability and optimizing drug cutaneous penetration, as illustrated by several examples. For instance, modification of established calcipotriol formulations with DSPE-PEG (1,2-Distearoyl-sn-glycero-3-Phosphoethanolamine-N-Polyethylene-glycol) increased colloidal stability and deposition of the drug in the stratum corneum, particularly with Small Unilamellar Vesicles (SUVs).^94^ Several additional experimental and clinical evaluations have been conducted with liposomal systems:

• Cationic liposomes containing cyclosporine reduced proinflammatory cytokines (TNF-α, IL-17, and IL-22) and improved clinical symptoms.^93^
• Systems incorporating ginsenoside Rg3 into microneedles prolonged cutaneous retention and reduced inflammation and epidermal thickening.^70^
• Formulations containing omiganan demonstrated controlled release and improved permeation, whereas mannosylated liposomes containing celastrol enhanced cellular uptake and exerted an antimaturation effect on dendritic cells.^93^
• Liposomes with glabridin and, separately, with the combination of ibrutinib-curcumin reduced psoriasis severity and inflammatory cytokines in a dose-dependent manner and exhibited a favorable safety profile.^93^

Solid Lipid Nanoparticles (SLNs) consist of a solid, biocompatible lipid matrix that can incorporate drugs, thereby increasing their solubility, stability, skin penetration, and circulation time, while reducing enzymatic degradation. Several formulations employing this nanocarrier have shown benefits in the topical treatment of psoriasis. For example, SLNs containing methotrexate demonstrated controlled release, higher skin permeation (≈80%), greater dermal deposition, and suppression of keratinocyte proliferation, with superior efficacy compared with conventional formulations. When applied to cyclosporine, SLNs promoted localized, controlled release in the skin, enhanced bioavailability, and reduced cytotoxicity. Hydrogel formulations containing leflunomide improved photostability and reduced skin irritation, whereas SLNs loaded with apremilast provided prolonged release, increased dermal retention, and high cellular internalization.^95^ Taken together, this body of evidence suggests that SLNs represent a promising platform for targeted topical delivery of therapeutic agents in psoriasis, increasing efficacy while minimizing systemic adverse effects.

Nanostructured Lipid Carriers (NLCs)^93^ have also been investigated experimentally:

• NLCs containing luteolin showed improved permeation, prolonged release (36 h), and reductions of TNF-α, IL-6, IL-17, and IL-23 levels in skin and blood.
• A nanogel combining tacrolimus and thymoquinone exhibited synergistic therapeutic potential and reduced toxicity.
• NLCs loaded with riluzole provided prolonged release, non-angiogenic activity, and inhibition of keratinocyte proliferation.
• Encapsulation of cannabidiol in NLCs increased chemical stability, reduced cytotoxicity, and enhanced anti-inflammatory effects.
• NLCs containing tazarotene and calcipotriol showed sustained release, minimal systemic absorption, and improved antipsoriatic efficacy.
• Co-formulation of fluocinolone acetonide and acitretin in NLCs reduced adverse effects and increased skin deposition compared with the conventional gel.

Thus, a wide range of nanoparticles is expected to facilitate the penetration of substances such as corticosteroids, tacrolimus, small-molecule drugs, and several other agents with established potential in the management of dermatoses, maybe increasing their efficacy while reducing adverse effects, particularly potential systemic toxicity, as well as overall costs for healthcare systems and for patients.

Another emerging and promising area is the use of nanotechnology in the treatment of skin cancer. Functionalized gold nanoparticles have been tested in clinical studies for photothermal therapy of basal cell and squamous cell carcinomas, selectively heating and destroying tumor cells after infrared laser activation.^96^ Gene therapies using topical nanocarriers are also emerging. For example, lipid nanoparticles loaded with anti-IL-17 and anti-TNF-α siRNAs are being evaluated in patients with psoriasis to modulate the local immune response in psoriatic lesions. Similarly, a nanoparticulate complex carrying two siRNAs has shown encouraging results in a phase II trial for cutaneous basal cell carcinoma.^97^

Finally, wound healing is a highly orchestrated process encompassing hemostasis, inflammation, proliferation, and remodeling. Disruption of these phases, as seen in chronic wounds, burns, or post-surgical scars, leads to persistent inflammation, excessive fibroblast activity, and disorganized extracellular matrix deposition, culminating in hypertrophic scars and keloids. Nanotechnology-based strategies provide a promising toolbox to modulate this microenvironment by enabling localized, sustained delivery of antimicrobials, antioxidants, growth factors, and anti-fibrotic agents. Nanostructured dressings, metal nanoparticle-loaded hydrogels, biomimetic scaffolds, and dissolvable microneedle patches can enhance re-epithelialization, control infection, and guide collagen remodeling, thereby improving both functional recovery and aesthetic outcomes.^14^, ^69^

## Safety aspects of nanotechnology in topical actives

From a translational standpoint, the clinical adoption of nano-enabled dermatologic products must go beyond proof-of-concept efficacy to address batch-to-batch reproducibility, manufacturing robustness, and effectiveness. Regulatory classification as a cosmetic, drug, or borderline product critically shapes the depth of safety and efficacy data required, as well as labeling and postmarketing surveillance obligations. Harmonized frameworks that integrate physicochemical characterization, in vitro and in vivo testing, human clinical data, and real-world registries will be essential to ensure that nanocosmeceuticals transition from promising prototypes to reliable, patient-centered therapeutic tools.

As nanotechnology enables greater permeation of active ingredients, it is imperative to ensure their safety during absorption and to assess their toxicity. The safety of nanotechnology in dermatology relies on a comprehensive characterization dossier and toxicological testing specifically tailored for nanomaterials. Essential descriptors include morphology, aggregation state, surface area, charge, solubility, and coatings. These factors determine how nanoparticles behave in formulations, interact with the skin, and affect bioavailability. Consistent and precise physicochemical characterization is crucial to reduce variability in safety studies, as emphasized by recent guidelines.^98^

With regards to cutaneous exposure, regulatory evidence is robust for nano-sized inorganic filters used in photoprotection. For titanium dioxide (TiO_2_, nano), the Scientific Committee on Consumer Safety has assessed that its use as a UV filter at concentrations up to 25% on intact skin is safe. However, caution is advised for inhalation due to potential respiratory risks. Zinc oxide (ZnO, nano) is similarly regarded; its use in sunscreen is considered safe, as particles remain primarily within the upper skin layers and systemic absorption is low. Surface coatings are recommended to prevent oxidative stress. The European regulatory framework distinguishes between dermal and inhalation safety and upholds stringent requirements for coatings.^99^

Regarding nanocarriers such as liposomes, solid lipid nanoparticles, and polymeric systems, safety primarily depends on excipients and surfactants, surface charge, and release behavior rather than the nano designation. Well-constructed formulations may reduce irritation by delivering lower doses of actives more effectively to the epidermis. Nonetheless, there are potential risks from cutaneous retention or impurities if manufacturing quality is subpar. Literature on LNPs and lipid systems emphasizes the importance of testing for phototoxicity, sensitization, and subclinical inflammation. Ultimately, appropriate design and quality control can mitigate or amplify potential risks.^52^

Finally, risk assessments must unite efficacy with realistic exposure scenarios. Topical nano-sized filters, for example, have not demonstrated consistent evidence of clinically relevant systemic absorption under standard conditions or carcinogenicity when properly formulated. Their cosmetic and protective benefits are clear, provided product integrity is maintained. Beyond UV filters, anti-aging and cosmeceutical applications require safety programs that consider vulnerable skin populations, assess free and matrix-bound particles, and include clinical trials focusing on irritation, sensitization, and inflammation markers.^100^, ^101^

## Critical appraisal of technologies

The use of nanocarriers is now a reality for dermatologists, who, as prescribers and researchers, play a central role in the critical assessment of these formulations. They must understand physicochemical parameters, characterization methods, and safety limitations before incorporating such products into clinical practice. Numerous options are being developed and show substantial experimental potential for clinical use in dermatoses; however, significant gaps remain to be addressed.

The authors describe here key recommendations for evaluating products that use nanoparticles, including:

• Methodology and reproducibility of studies: It is recommended that reports include particle size, polydispersity index, zeta potential (a measure of the degree of electrostatic repulsion between charged particles in suspension), encapsulation efficiency, release profile, accelerated stability, and methods for quantifying cutaneous deposition (e.g., tape stripping, microdialysis).
• Clinical trials: priority should be given to active comparators, clinically meaningful endpoints beyond tolerability and quality of life, cost-effectiveness analyses, and long-term safety, with prior trial registration and transparency regarding negative data to mitigate bias.
• Regulation and labelling: harmonization of criteria for “nano” labelling is needed, with clear disclosure for consumers and professionals; FDA guidance and ANVISA initiatives for risk and biopersistence assessment should be considered.
• Databases and traceability: the creation of national and international registries to monitor adverse events and clinical effectiveness of nano-structured products is advisable to strengthen pharmacovigilance.

## Final considerations

The authors acknowledge the importance of critically evaluating individual study quality. However, the expansive and heterogeneous literature, ranging from preclinical experiments to clinical trials, presents significant challenges to standardized assessment within a single manuscript. A formal study-by-study appraisal would necessitate a systematic review, which was not the intent of this article. Instead, this narrative approach identifies overarching patterns and translational gaps, serving as a foundation for future systematic evaluations of specific nanodermatology questions.

The past decade has consolidated clinical nanodermatology, with successful proof-of-concept studies demonstrating improved therapeutic efficacy and reduced adverse effects. Promising areas include combining nanocarriers with established therapies and developing biofunctional nanotechnologies capable of responding to skin microenvironmental stimuli for controlled drug release. Despite advances, challenges remain in the long-term safety of nanoparticles, industrial scalability, and the need for larger multicenter trials. Nonetheless, cutaneous nanomedicine holds a promising perspective for advancing dermatologic management, particularly for chronic and refractory diseases. The development of stimuli-responsive systems designed to modulate drug release based on physiological cues represents a move toward more personalized and precise cutaneous therapies.

Only a limited number of nano-enabled formulations have undergone systematic evaluation in human clinical trials; most studies remain small, single-centre, and exploratory. Best-characterized products include liposomal and vesicular systems for inflammatory dermatoses, inorganic UV filters, nanostructured moisturizers, and nanoemulsion formulations for pigmentary disorders, demonstrating favorable efficacy and tolerability. However, heterogeneity in trial design, short follow-up periods, and absence of active comparators limit the strength of conclusions and preclude robust comparisons with current standards of care, as discussed above. Future research should prioritize adequately powered, multicentre randomized trials with standardized endpoints, including disease severity scores, imaging-based measures, and quality-of-life indices, together with long-term safety monitoring. This evidence base is essential to support rational incorporation of nanoformulations into routine practice and distinguish therapeutic advantages from marketing-driven claims.

Continuous development of nanoparticles with improved physicochemical properties and greater loading capacity is expanding therapeutic and cosmetic possibilities, contributing to more rational, effective, and economically sustainable dermatologic practice. These platforms enable precise, multifunctional drug delivery, potentially safer, more effective, and more personalized therapies suitable for sensitive skin types. Nevertheless, a deeper understanding of nanomaterial-skin interactions and the effects of metabolites and residuals on human health and the environment remains essential. The role of nanotechnology in modern dermatology is well-established, provided it is accompanied by methodological rigor, ongoing safety surveillance, and transparent regulation. Advances in artificial intelligence and digital dermatology are expected to accelerate rational design and clinical optimization of nano-enabled therapies by integrating skin imaging, omics, and patient-reported outcomes to tailor formulations to individual needs.

## ORCID ID

Clarissa Prati Bernardi Cogo: 0000-0002-7279-1126

Mayra Ianhez: 0000-0003-3604-3128

Renata Bertino: 0009-0008-6887-7956

Anderson Alves Costa: 0000-0003-0504-2472

Adilson Costa: 0000-0003-0873-6840

Hélio Amante Miot: 0000-0002-2596-9294

## Declaration of generative AI in scientific writing

During the preparation of this work, the authors used ChatGPT 5.2 to assist with English-language editing. After using this tool, the authors reviewed and edited the content as needed and take full responsibility for the content of the published article.

## Statement of original publication

This is an original and unpublished work and is not under simultaneous consideration for publication elsewhere.

## Financial support

The authors received financial support from Lecieza Dermocosmetics for the preparation of this manuscript. Lecieza Dermocosmetics had no role in the study design, data collection, analysis, or manuscript preparation. CNPq (306358/2022-0) – Hélio Amante Miot is a CNPq research fellow.

## Authors' contributions

Clarissa Prati: Drafting of the manuscript; preparation of illustrative figures; critical review of the literature; final approval of the manuscript.

Mayra Ianhez: Drafting of the manuscript; critical review of the literature; final approval of the manuscript.

Renata Bertino: Drafting of the manuscript; critical review of the literature; final approval of the manuscript.

Anderson Costa: Drafting of the manuscript; critical review of the literature; final approval of the manuscript.

Adilson Costa: Drafting of the manuscript; critical review of the literature; final approval of the manuscript.

Hélio Amante Miot: Drafting of the manuscript; critical review of the literature; final approval of the manuscript.

## Research data availability

The entire dataset supporting the results of this study was published in this article.

## Conflicts of interest

Clarissa Prati: Advisory Board – Johnson & Johnson, Sanofi, UCB-Biopharma, AbbVie, Megalabs, Novartis, Lecieza; Speaker – Johnson & Johnson, Sanofi, UCB-Biopharma, AbbVie, Megalabs, Novartis, Pierre-Fabre, Boehringer-Ingelheim, Libbs, Cristália, Mantecorp, Leo Pharma, Lilly, Lecieza; Clinical research – Sanofi, Pierre-Fabre, Lilly, Celldex.

Mayra Ianhez: Advisory Board – Galderma, Sanofi, Pfizer, Novartis, AbbVie, Johnson & Johnson, UCB-Biopharma, Boehringer-Ingelheim; Speaker – Galderma, Sanofi, Pfizer, Theraskin, Novartis, AbbVie, Janssen, Leo Pharma, FQM.

Renata Bertino: No conflicts of interest were declared.

Anderson Alves Costa: Advisory Board – Lilly, Johnson & Johnson, Novartis, Pfizer; Speaker – AbbVie, Eli Lilly, Janssen, Novartis, Pfizer, Sanofi, and UCB Pharma.

Adilson Costa: No conflicts of interest were declared.

Hélio Miot: Advisory Board – Johnson & Johnson, L’Oréal, Theraskin, Sanofi, Pfizer; Clinical research – AbbVie, Pierre-Fabre, Galderma, Eucerin, Theraskin, Merz.
