# Supplementary material for: Nanotechnology in dermatologic therapy: clinical advances and emerging translational perspectives over the past decade
Source: An Bras Dermatol. 2026 Aug 6;101(5):501427. doi: 10.1016/j.abd.2026.501427 (PMC13471706; doi:10.1016/j.abd.2026.501427)
Supplement: Supplementary file 1 [file mmc1.docx]

ABD-D-26-00208_Supplementary Material

**Supplementary Table 1** Examples of dermocosmetic products identified as having a declared or documented use of nanotechnology over the past 10-years, including international and Brazilian product lines.

| **Product** | **Manufacturer** | **Category** | **Key actives** | **Type of nanosystem** | **Class** | **Year** | **Market** |
| --- | --- | --- | --- | --- | --- | --- | --- |
| Reduxcel Nano Firm | Adcos | Body firming product | CitoDetox; taurine; firming complexes | No public disclosure | Multifunctional | nan | Brazil |
| Ivy C AOX Gel | Mantecorp Skincare | Facial rejuvenating gel | Nanoencapsulated vitamin C; hyaluronic acid; (some communications: retinol nanospheres) | Liposome | Anti-aging / Skin-renewing | nan | Brazil |
| Mineral Sunscreen Fluid SPF 50+ | Avène | Mineral sunscreen | Titanium dioxide and zinc oxide | No public disclosure | Photoprotection | nan | EU / Global |
| Fluido Mineral SPF 50+ | Avène | Mineral sunscreen | Titanium dioxide and zinc oxide | No public disclosure | Photoprotection | nan | EU / Global |
| Photoderm Mineral Fluide SPF 50+ | Bioderma | Mineral sunscreen | Titanium dioxide and zinc oxide | No public disclosure | Photoprotection | nan | EU |
| Bariésun Mineral Cream SPF 50+ | Uriage | Mineral sunscreen | Titanium dioxide and zinc oxide | No public disclosure | Photoprotection | nan | EU / Global |
| Mesoprotech Mineral Fluid SPF 50+ | Mesoestetic | Mineral sunscreen | Titanium dioxide, zinc oxide; anti-aging actives | No public disclosure | Photoprotection | nan | EU / Global |
| Photoprotector Pediatrics Fusion Fluid Mineral Baby SPF 50 | ISDIN | Mineral sunscreen | 100% mineral filters (titanium dioxide, zinc oxide) | No public disclosure | Photoprotection | nan | EU / Global |
| UV Clear Broad-Spectrum SPF 46 | EltaMD | Mineral sunscreen | Zinc oxide 9% (micronized), octinoxate 7.5% | Metal oxide (titanium dioxide, nano zinc oxide) | Photoprotection | nan | USA and other markets |
| Photoprotector Fusion Fluid Mineral SPF 50 (Face) | ISDIN | Mineral facial sunscreen | Zinc oxide; titanium dioxide (100% mineral filters; formulations vary by country) | No public disclosure | Photoprotection | nan | Brazil / EU / Global |
| Anthelios Mineral Zinc Oxide Sunscreen SPF 50 | La Roche-Posay | Mineral facial sunscreen | Zinc oxide; titanium dioxide; antioxidants (Cell-Ox Shield^®^) | Metal oxide (titanium dioxide, nano zinc oxide) | Antioxidant / Rejuvenating | nan | Global / EU, USA, Brazil |
| Sun Sensitive Mineral Sunscreen Lotion SPF 50 | Eucerin | Mineral sunscreen (face/body) | Zinc oxide | Metal oxide (titanium dioxide, nano zinc oxide) | Photoprotection | nan | USA / Global |
| Mesoprotech Mineral Fluid SPF 50+ | Mesoestetic | Tinted mineral sunscreen | Zinc oxide; titanium dioxide (exclusively mineral filters) | No public disclosure | Photoprotection | nan | EU / Global |
| Protoderm Nude Touch Mineral SPF 50+ | Bioderma | Tinted mineral sunscreen | Zinc oxide | Metal oxide (titanium dioxide, nano zinc oxide) | Photoprotection | nan | EU / Global |
| Anthelios Mineral Tinted SPF 50 | La Roche-Posay | Tinted mineral sunscreen | Zinc oxide; titanium dioxide | Metal oxide (titanium dioxide, nano zinc oxide) | Photoprotection | nan | Global |
| C-VIT 5 Liposomal Serum | Sesderma | Antioxidant serum | Five forms of vitamin C; antioxidants | Liposome | Antioxidant / Rejuvenating | nan | Global |
| C-VIT Liposomal Serum | Sesderma | Antioxidant serum | Vitamin C (mixture of derivatives), ferulic acid, hyaluronic acid | Liposome | Antioxidant / Rejuvenating | nan | Global |
| Azelac RU Liposomal Serum | Sesderma | Depigmenting serum | Azelaic acid, 4-butylresorcinol, niacinamide (according to local labelling) | Liposome | Depigmenting / Brightening | nan | Global |
| Copper Peptide (CAIS/CAIL) | NIOD (DECIEM) | Repair serum | Copper peptides | No public disclosure | Anti-aging / Skin-renewing | nan | Global |
| Anthelios Mineral One SPF 50+ | La Roche-Posay | Tinted mineral sunscreen | Zinc oxide; titanium dioxide | Metal oxide (titanium dioxide, nano zinc oxide) | Photoprotection | 2020–2021 | EU / Global |
| Capital Soleil Mineral Tinted SPF 50+ | Vichy | Tinted mineral sunscreen | Zinc oxide; titanium dioxide | No public disclosure | Photoprotection | 2019–2021 | EU / Global |
| Protect AC Mineral SPF 50+ | A-Derma | Mineral sunscreen | Zinc oxide; titanium dioxide | No public disclosure | Photoprotection | 2018–2020 | EU / Global |
| Bariésun Mineral Stick SPF 50+ | Uriage | Mineral sunscreen stick | Zinc oxide; titanium dioxide | No public disclosure | Photoprotection | 2019–2022 | EU / Global |
| Photoderm Mineral Spray SPF 50+ | Bioderma | Mineral sunscreen spray | Zinc oxide; titanium dioxide | No public disclosure | Photoprotection | 2018–2020 | EU |
| Eryfotona AK-NMSC Mineral SPF 100+ | ISDIN | Mineral photoprotector for adjunctive prevention | Zinc oxide; DNA Repairsomes^®^ (encapsulated photolyase) | No public disclosure | Photoprotection | 2018–2021 | EU / USA / Brazil |
| Sensitive Mineral Face Sunscreen SPF 50 | Eucerin | Mineral facial sunscreen | Zinc oxide | Metal oxide (titanium dioxide, nano zinc oxide) | Photoprotection | 2020–2022 | USA / EU |
| Melan tran3x Gel-Cream | mesoestetic | Depigmenting gel-cream | Tranexamic acid, niacinamide, mandelic acid | No public disclosure | Depigmenting / Brightening | 2018–2020 | Global |
| Retises 0.5 Regenerating Cream | Sesderma | Rejuvenating cream | Retinol; retinaldehyde; antioxidants | Liposome | Anti-aging / Skin-renewing | 2018–2022 | Global |
| Concentrado Vitamina C 20% | Adcos | Antioxidant serum | Stabilized vitamin C; ferulic acid; hyaluronic acid | No public disclosure | Antioxidant / Rejuvenating | 2019–2022 | Brazil |
| Vitamina C 10 Aquagel | Mantecorp Skincare | Antioxidant gel | Vitamin C derivatives; hyaluronic acid | No public disclosure | Antioxidant / Rejuvenating | 2019–2021 | Brazil |
| Tranexamic Acid Liposomal Serum | Sesderma (Azelac RU) | Depigmenting serum | Tranexamic acid; niacinamide; kojic acid (varies by market) | Liposome | Depigmenting / Brightening | 2020–2023 | Global |
| Sérum Antissinais Lecieza | Lecieza Dermocosmetics | Anti-aging serum | Nano vitamin C 10%; nano peptide; polyglutamic acid; carnosine | Polymeric nanocapsule | Anti-aging / Skin-renewing | 2024–2025 | Brazil |
| Sérum Antioxidante Lecieza | Lecieza Dermocosmetics | Antioxidant serum | Astaxanthin; nano vitamin C 10%; nano resveratrol; nano quercetin | Polymeric nanocapsule | Antioxidant / Rejuvenating | 2024–2025 | Brazil |
| Sérum Clareador Lecieza | Lecieza Dermocosmetics | Brightening serum | Nano niacinamide; glycolic acid; alpha-arbutin; nano retinyl palmitate | Polymeric nanocapsule | Depigmenting / Brightening | 2024–2025 | Brazil |
| Sérum Antiacne Lecieza | Lecieza Dermocosmetics | Anti-acne serum | Nano salicylic acid; nano bakuchiol; nano hyaluronic acid; plant polysaccharides | No public disclosure | Anti-acne / Sebum-regulating | 2024–2025 | Brazil |

Source: Authors’ compilation based on publicly available data and official brand websites (2020–2025). Legend: nan (no specific launch year available), EU (European Union), USA (United States of America).
